# Dreaming and Sleep-Related Metacognitions in Patients with Sleep Disorders

**DOI:** 10.3390/clockssleep4030034

**Published:** 2022-09-01

**Authors:** Michael Schredl, Claudia Schilling

**Affiliations:** Sleep Laboratory, Central Institute of Mental Health, Medical Faculty Mannheim, Heidelberg University, 68159 Mannheim, Germany

**Keywords:** sleep-related metacognitions, dreaming, nightmares, emotional tone of dreams

## Abstract

Sleep-related metacognitions play a role in the etiology of insomnia and are distressing while falling asleep. Although similar concepts, such as thought suppression, have been studied in the context of dreaming, the relationship between sleep-related metacognitions and more negatively toned dreaming due to stressful pre-sleep experiences has yet to be studied. Overall, 919 patients with various sleep disorders completed the Metacognitions Questionnaire-Insomnia (MCQ-I20), Arousal Disposition Scale (APS), and Pre-Sleep Arousal Scale (PSAS) and kept a sleep diary over seven days eliciting dream recall, nightmare frequency, and the emotional tone of their dreams. The regression analysis showed that the MCQ-I20 (small effect size) and the APS (medium effect size) were associated with nightmare frequency and negatively toned dream emotions. These findings suggest that dysfunctional sleep-related metacognitions that are active prior to sleep are also associated with more negatively toned dreaming and more nightmares—even after controlling for trait arousability. It would be very interesting to study where therapeutic strategies, such as metacognitive therapy explicitly targeting sleep-related metacognition, could also be beneficial with regard to dreams (more positive dreams and fewer nightmares).

## 1. Introduction

Sleep-related metacognitions play a role in the etiology of insomnia [1], that is, dysfunctional metacognitions, such as, “Before I fall asleep, I should try and switch off my thoughts.” or “Before I fall asleep, I should try as many ways as I can to control my thoughts.” are much more frequent in patients with insomnia compared to healthy sleepers [2,3,4] and patients with other sleep disorders [5]. Based on the continuity hypothesis of dreaming, which states that dream content reflects waking-life experience [6], one would expect that dysfunctional metacognitions that occur at sleep onset and cause stress for the affected person might affect subsequent dream content in a negative way. However, studies directly linking sleep-related metacognitions with dreaming have not yet been carried out. Indirect evidence is provided by studies showing that patients with insomnia experience more negatively toned dreams [7,8,9] and more nightmares [10], as these patients more often report dysfunctional sleep-related metacognitions [2,3,4]. This might, however, also be explained by heightened stress levels in the patient group affecting metacognitions and dreaming, especially nightmares [11].

Within the field of dream research, several studies [12,13,14,15] have examined the so-called “dream rebound effect”: persons who are instructed to suppress specific thoughts prior to sleep onset are more likely to dream about it. Malinowski, Carr, Edwards, Ingarfill, and Pinto [15] were able to demonstrate that thought suppression of negatively toned thoughts showed an even stronger dream rebound effect, i.e., it occurred more often in dreams, than for the suppression of positive thoughts. Persons who show high trait thought suppression, as measured with the White Bear Suppression Inventory WBSI [16], dreamed more often about their waking-life emotions [17] and typically occurring worries in this study than persons with low trait thought suppression. More negatively toned dreams were also found in the high thought suppressor group [18]. A closer look at items of the WBSI [16] reveals interesting similarities to the MCQ-I [2], e.g., “Sometimes I wish I could stop thinking.” or “I wish I could stop thinking of certain things”. In addition, thought suppression is also related to poorer sleep quality [18,19]. Intrusive thoughts, whether suppressed or focused upon, are also followed by more negatively toned dreams [20]; that is, pre-sleep rumination also affects dreams in a negative way [21]. Taken together, these empirical data would predict that dysfunctional sleep-related metacognitions are associated with negatively toned dreams.

The present study addressed the question of whether patients with frequent sleep-related metacognitions experience negatively toned dreams and nightmares more often. Based on the continuity hypothesis [6], we expected a positive relationship—even after controlling for the type of sleep disorder (e.g., insomnia). Since trait aspects (affect distress) play an important role in nightmare etiology [11], we also wanted to know whether arousal disposition, that is, being affected by stress, might explain—at least partly—the relationship between sleep-related metacognitions and negatively toned dreaming.

## 2. Method

### 2.1. Participants

Overall, 919 patients (542 women, 377 men) were included in the present study. Their mean age was 44.88 ± 17.22 years. The diagnoses and probable diagnoses are presented in Table 1. The two largest groups are patients with insomnia or probable insomnia and patients with RLS or PLMD or probable RLS or PLMD. Due to the sleep laboratory being located in a psychiatric clinic and not offering CPAP treatment, the number of persons with sleep-related breathing disorders or probable sleep-related breathing disorders was relatively small. A substantial number of persons with hypersomnia and parasomnia diagnoses were also part of the patient group. About a third of the patients had a primary mental disorder, such as mood disorder, anxiety disorder, eating disorder, schizophrenia, addictions syndromes, and borderline personality disorder. Other sleep disorders included insufficient sleep syndrome, probable insufficient sleep syndrome, shift work-related sleep disorders, and sleep-related rhythmic movement disorders. Somatic syndromes that were directly associated with sleep impairment were chronic pain syndromes, epilepsy, Parkinson’s disease, dementia, etc.

### 2.2. Measurement Instruments

The sleep diary [22] included 16 items per day and was to be kept over a seven day period. The items assess sleep behavior, e.g., bedtime, sleep latency, and several dream variables, which were included in the present analysis. For measuring dream recall, the following response options were presented: no dream recall, impression of having dreamed without remembering any specific dream content, and successful dream recall. Participants could—if they wanted to do so—record the overall theme of the dream in a few words. The overall emotional tone of the dream was measured with a five-point scale: −2 = strong negative emotions, −1 = negative emotions, 0 = balanced emotions/neutral, +1 = positive emotions, and +2 = strong positive emotions. Lastly, the participants were asked whether the dream was a nightmare (Yes/No).

The Metacognitions Questionnaire-Insomnia (MCQ-I) was originally developed by Waine, Broomfield, Banham, and Espie [2] and includes 60 questions about sleep-related metacognitions. A German short form of the questionnaire with 20 items, the MCQ-I20, was developed by Schredl et al. (2021). Each statement should be answered on a four-point Likert scale ranging from “Do not agree (1)” to “Mainly agree (4)”; for example, “Before I fall asleep, I should try and switch off my thoughts.” or “Not being able to rest my mind in bed means I’m abnormal”. The MCQ-I20 showed high internal consistency (Cronbach’s alpha = 0.906) and, for an average retest interval of 150 days, the retest reliability was high (r = 0.916, *n* = 19) [5]. In addition, the correlation between the score of the full questionnaire (60 items) and the sum score of the 20 items was very high: r = 0.953, *p* < 0.0001, *n* = 458 [5].

The Arousal Predisposition Scale (APS) consists of twelve five-point Likert items and measures individual differences of arousal predisposition (trait arousal) [23]. The categories ranged from never (1) to always (5), e.g., “I get excited easily.”, “I get flustered if I have several things to do at once.”, or “Sudden changes of any kind produce an immediate emotional effect on me.” A total sum score is calculated over all items with one item (“I am a calm person”) reversed. Higher scores indicate higher trait arousability. The split-half reliability was α = 0.83 [24]. We used a German translation of the APS that is very similar to the translation of Clamor et al. [25].

The original Pre-Sleep Arousal Scale (PSAS) includes 16 five-point Likert scales (ranging from 1 = not at all to 5 = extremely) for eliciting possible symptoms of arousal at bedtime the previous night, i.e., it is a state measure [26]. Eight items were aimed at assessing cognitive arousal (e.g., “Can’t shut off your thoughts”), and eight items for somatic arousal (e.g., “jittery and nervous feeling in the body”). Higher scores indicate higher pre-sleep arousal. A German version was created and evaluated by Gieselmann et al. [27], dropping one item of the cognitive arousal scale as it loaded on both factors. The internal consistency of the German version was high for both scales: the cognitive arousal scale α = 0.94 and the somatic arousal scale α = 0.80. The PSAS scales differentiated well between good and bad sleepers [27].

### 2.3. Procedure

The patients were referred to the sleep center by physicians, psychiatrists, and neurologists working in the ambulatory sector of the German health system for evaluating sleep problems. The sample included all patients referred from February 2019 to February 2022. For most of the patients, the primary complaint was sleep disorder(s), but a substantial number of patients with mental disorders were also referred, as the sleep problems did not subside under standard therapy. All patients were seen by a certified sleep specialist for about 30 to 45 min. The diagnoses were based on the clinical interview in accordance with the ICD-10 [28]. The sleep diary and the three questionnaires were sent to the patients prior to their appointment, asking them to bring in the completed 7-day diary and the questionnaires. As the sleep diary and the questionnaires were part of the clinical routine, almost all patients returned the materials; the percentage of missing data is very small (less than 5%), mainly due to language problems (non-German speakers).

Dream recall frequency based on the diary was calculated as days per week with successful dream recall. For persons with missing values, that is, less than 7 days of recording, the mean recall rate per day was computed and multiplied by 7. In order to obtain an ordinal scale, only integer numbers (zero to seven) were allowed and rounded if necessary. Ordinal scaling was necessary, as dream recall frequency was not normally distributed. The Yes/No answers for the nightmares were averaged and then multiplied by 30 to obtain the units per month. The nightmare frequency was also not normally distributed, and the participants were categorized into five groups: 0 = no nightmares, 1 = 1 to 5 nightmares per month, 2 = 6 to 10 nightmares per month, 3 = 11 to 20 nightmares per month, and 4 = more than 20 nightmares per month. The emotional tone of the dreams was averaged and treated as an interval scale, as it was normally distributed around the mean of zero. For descriptive purposes, means and standard deviations (M ± SD) were used. We computed correlations and regression analyses according to the levels of the scales, e.g., Pearson correlations or Spearman rank correlations, as well as parametric or ordinal regression. For the effects of the APS and MCQ-I20 on the dream variables, we computed effect sizes according to Cohen [29] using the website of Lenhard and Lenhard [30].

## 3. Results

The mean number of mornings with valid data for dream recall was 6.32 ± 1.50. For all participants, the mean dream recall was 1.95 ± 2.29 mornings per week. The distribution of the rounded dream recall (only integers allowed) is depicted in Table 2. More than 40% of the participants did not recall a dream within the diary period, but a substantial number (about 17%) recalled dreams on at least five mornings per week. The mean number of mornings with valid data for the nightmare item was 6.02 ± 1.68; there are slightly more missing values compared to that of dream recall. The mean nightmare frequency was 3.41 ± 6.94 nightmares per month; the nightmare frequency distribution is depicted in Table 3. The majority of participants (70%) did not report nightmares within the diary period, however, about 19% reported five or more nightmares per month. The mean number of ratings regarding the emotional tone of dreams was 2.95 ± 2.63. This is higher than the number of successful dream recalls, as some ratings were associated with the impression of having dreamed but with no explicit recall. The average emotional tone was −0.37 ± 0.86 (*n* = 653); the dreams of this patient group were, on average, more often negative than positive.

Table 4 shows the means and standard deviations of the four scales: Metacognitions Questionnaire-Insomnia (MCQ-I20); Arousal Predisposition Scale (APS); Pre-Sleep Arousal Scale (PSAS), Somatic Arousal; and Pre-Sleep Arousal Scale (PSAS), Cognitive Arousal. The inter-correlation between the scales were as follows: r = 0.393 (MCQ-I20—APS), r = 0.397 (MCQ-I20—PSAS-S), r = 0.581 (MCQ-I20—PSAS-C), r = 0.433 (APS—PSAS-A), r = 0.418 (APS—PSAS-C), and r = 0.572 (PSAS-S—PSAS-C), all *p* < 0.0001. The Arousal Predisposition Scale (APS) was the only scale to correlate with dream recall frequency (see Table 4). All four scales were associated with nightmare frequency and the dreams’ emotional tone; that is, higher scores of the waking variables were reflected in higher nightmare frequency and more negatively toned dreams (see Table 4).

The regression analyses for the three dream variables are depicted in Table 5. In addition to the covariates age, gender, and diagnoses, the Arousal Predisposition Scale (APS) and the Metacognitions Questionnaire-Insomnia (MCQ-I20) were entered simultaneously into the analyses. The Arousal Predisposition Scale (APS) was associated with dream recall frequency (effect size = 0.254), whereas the Metacognitions Questionnaire-Insomnia (MCQ-I20) was not (effect size = 0.102). As expected, the MCQ-I20 and the APS were associated with nightmare frequency; the APS shows a stronger effect (d = 0.249) than the MCQ-I20 (d = 0.124). Furthermore, both scales were associated with negative dream emotions, and, again, the APS shows a stronger association (d = 0.425 than the MCQ-I20 (d = 0.291). Age was associated with dream recall (less dream recall in older persons) and with nightmare frequency (see Table 5). In addition, women tended to recall dreams more often than men in this sample. Older persons also reported slightly more positively toned dreams than younger persons. As expected, patients with a diagnosis of a nightmare disorder recalled more dreams and more nightmares, and their dream emotions were more negatively toned compared to patients without a nightmare disorder. The other significant findings should be viewed with caution as the presence or absence of a diagnosis does not compare this group with healthy controls, but with the other group of patients who also suffer from one sleep disorder or another.

## 4. Discussion

In a large sample of patients with sleep disorders, dysfunctional sleep-related metacognitions were related to negative dreaming and nightmares, even after controlling for trait arousability and the presence/absence of a specific sleep disorder diagnosis. We also found a negative association between the two subscales of the Pre-Sleep Arousal Scale (they were correlated with trait arousability and sleep-related metacognitions) and dreaming. In addition, trait arousability was related to dream recall frequency, supporting the arousal–retrieval model of dream recall proposed by Koulack and Goodenough [31].

Prior to discussing the findings in detail, a few methodological issues must be taken into consideration. The present sample consisted of patients with a broad spectrum of sleep disorders. This was necessary for the objective of the study since Palagini et al. [32] were able to demonstrate that good sleepers did not report any dysfunctional sleep-related metacognitions. In the present analyses, we accounted for this by including all diagnoses (confirmed and probable) as possible confounders into the regression analyses. The strongest effect on dream recall, nightmare frequency, and negative dream emotions was found for the patients with a nightmare disorder diagnosis, which is perfectly plausible. We refrained from interpreting smaller effects, as many tests have been performed, and studying the differences between patients with different sleep disorders is not that helpful, e.g., whether insomnia patients had more nightmares than patient with sleep-related breathing disorders. For these analyses, a control sample of healthy sleepers would be necessary; however, this was not the objective of the present study. The decrease in dream recall and nightmare frequency with age fits nicely with other large-scale studies, e.g., [33], and, thus, provides support for the validity of the present findings. Similarly, the gender difference in dream recall is also in accordance with a meta-analysis [34], but we did not find a gender difference in nightmare frequency [35]; this might be explained by the fact that patients with sleep disorders more often experience nightmares than healthy controls [36].

Dream recall frequency and nightmare frequency was measured with a sleep diary (mainly focusing on sleep behavior and sleep quality), which is considered by researchers, e.g., [37], to be more appropriate than retrospective scales. However, there are pros (minimizing recall biases) and cons (increasing dream recall and nightmare frequency by focusing the participants’ attentions towards dreaming by keeping the diary) [38,39], and Zunker, Althoff, Apel, Lässig, Schültke, and Schredl [39] showed that differences in nightmare frequencies using retrospective measures versus diaries are quite small (effect size = 0.101); that is, the retrospective respective the prospective bias might not be that strong.

The diagnoses were based on the ICD-10 as this is obligatory in the German healthcare system. This might explain the high number of probable insomnia diagnoses, as it is recommended to code F51.0 only if the insomnia symptoms dominate [28]; that is, since periodic limb movement syndrome (PLMS) or a sleep-related breathing disorder can be the main source of the sleep problems, the insomnia diagnosis in the clinical interview prior to polysomnographic evaluation often cannot be given with certainty.

As expected, sleep-related metacognitions were related to negative dreaming in patients with sleep disorders independent of their diagnoses. Based on the continuity hypothesis [6], this relationship makes sense as these metacognitions result in problems when falling asleep and, thus, are distressful for the patients [1]. This distress is then reflected in more negatively toned dreams and nightmares. We were also able to demonstrate that trait arousability, which is also associated with negative dreams, does not fully explain the relationship; that is, metacognitions do not affect dreaming only in persons with high trait arousability.

As the present study is correlational in nature, it would be interesting to conduct experimental studies to back up these findings. It might be difficult to manipulate the extent of sleep-related metacognitions in patients with sleep disorders on a day-to-day basis (similar to the thought suppression experiments reviewed in the introduction), since these cognitive patterns typically develop over years. It would be interesting to investigate whether or not a specific therapeutic approach to reduce dysfunctional sleep-related cognitions by using MCT techniques [40] would also result in more positively toned dreams and fewer nightmares. The findings that trait arousability and pre-sleep arousal were associated with higher nightmare frequencies fits very well in the current models of nightmare etiology [11], indicating that both affect distress (trait) and affect load (state) contribute to nightmare occurrence; see also Schredl [41].

The finding that trait arousability was related to dream recall frequency supports the arousal–retrieval model of dream recall [31]; that is, dream recall is facilitated if the person experiences high arousal levels after waking up from a dream so that distractions cannot hamper the dream recall. The item of the APS “I tend to remain excited or moved for a long period of time after seeing a good movie.” [42] nicely illustrates this idea that the dream, defined as an experience during sleep [43], leads to prolonged arousal and better dream recall in persons with high trait arousability.

To summarize, the present findings suggest that dysfunctional sleep-related metacognitions that are active prior to sleep, that is, within the prolonged process of falling asleep, are also associated with more negatively toned dreaming and more nightmares. From a clinical viewpoint, it would be very interesting to study the extent to which cognitive behavioral therapy for insomnia [44], which has been shown to reduce dysfunctional sleep-related metacognitions [45], also improves dream emotions and reduces nightmares.

## Figures and Tables

**Table 1:** Diagnoses (multiple diagnoses possible) (*n* = 919).

Diagnoses	Frequency	Percent
Insomnia	231	25.14%
Insomnia (probable)	250	27.20%
Restless legs syndrome (RLS)/Periodic limb movement disorder (PLMD)	201	21.87%
Restless legs syndrome (RLS)/Periodic limb movement disorder (PLMD) (probable)	370	40.26%
Hypersomnia/Narcolepsy	31	3.37%
Hypersomnia/Narcolepsy (probable)	108	10.75%
Nightmare disorder	106	11.53%
REM sleep behavior disorder (RBD)	10	1.09%
REM sleep behavior disorder (RBD) (probable)	44	4.79%
NREM-Parasomnias	78	8.49%
NREM-Parasomnias (probable)	15	1.63%
Sleep-related breathing disorders (SRBD)	130	14.15%
Sleep-related breathing disorders (SRBD) (probable)	151	16.43%
Circadian rhythm sleep–wake disorders	44	4.79%
Mood disorders	216	23.50%
Other mental disorders	145	15.78%
Other sleep disorders	81	8.81%
Somatic illnesses closely related to sleep disorder	52	5.66%
No sleep disorder	5	0.54%

**Table 2:** Distribution of dream recall frequency (*n* = 919).

Mornings with Dream Recall	Frequency	Percent
7	77	8.38%
6	42	4.57%
5	41	4.46%
4	63	6.86%
3	85	9.25%
2	127	13.82%
1	98	10.66%
0	386	42.00%

**Table 3:** Distribution of nightmare frequency (*n* = 911).

Nightmares Per Month	Frequency	Percent
More than 20	45	4.94%
11 to 20	57	6.26%
6 to 10	72	7.90%
1 to 5	92	10.10%
0	645	70.80%

**Table 4:** Means, standard deviations, and zero-order correlations.

		Dream Recall			Nightmares			Emotional Tone of Dreams		
	Mean ± SD	r ^1^	*p*	*n*	r ^1^	*p*	*n*	r ^2^	*p*	*n*
Metacognitions Questionnaire-Insomnia (MCQ-I20)	41.59 ± 11.73 (855)	0.022	0.5186	855	0.136	<0.0001	848	−0.206	<0.0001	614
Arousal Predisposition Scale (APS)	35.66 ± 7.94 (896)	0.189	<0.0001	896	0.222	<0.0001	888	−0.258	<0.0001	639
Pre-Sleep Arousal Scale (PSAS), Somatic Arousal	13.78 ± 5.53 (855)	0.056	0.1048	855	0.160	<0.0001	847	−0.258	<0.0001	609
Pre-Sleep Arousal Scale (PSAS), Cognitive Arousal	16.89 ± 7.75 (859)	0.052	0.1247	859	0.157	<0.0001	851	−0.159	<0.0001	614

^1^ Spearman rank correlation, ^2^ Pearson correlation.

**Table 5:** Regression analyses for dream variables.

	Dream Recall ^1^			Nightmares ^1^			Emotional Tone of Dreams ^2^		
Variables	SE	Χ ^2^	*p*	SE	Χ ^2^	*p*	SE	t	*p*
Age	−0.1818	18.2	<0.0001	−0.2587	21.2	<0.0001	0.0884	2.0	0.0419
Gender (1 = f, 0 = m)	0.1264	10.8	0.0001	0.0477	0.9	0.3354	0.0286	0.7	0.4615
Insomnia	−0.0273	0.4	0.5190	−0.1163	4.5	0.0334	0.0632	1.5	0.1319
Insomnia (probable)	0.0073	0.0	0.8589	−0.1675	9.2	0.0024	0.1510	3.6	0.0003
RLS/PLMD	−0.0263	0.4	0.5243	0.0500	0.9	0.3425	0.0749	1.8	0.0760
RLS/PLMD (probable)	0.0235	0.3	0.5718	0.0270	0.3	0.6076	0.0420	1.0	0.3224
Hypersomnia/Narcolepsy	−0.0116	0.1	0.7524	−0.0241	0.3	0.5868	0.1270	3.3	0.0009
Hypersomnia/Narcolepsy (probable)	0.0502	1.6	0.2018	−0.0353	0.5	0.4605	0.0523	1.3	0.2064
Nightmare disorder	0.3155	71.3	<0.0001	0.4634	124.5	<0.0001	−0.2806	−7.2	<0.0001
RBD	−0.0173	0.2	0.6254	0.0552	2.1	0.1505	0.0736	2.0	0.0435
RBD (probable)	0.0766	4.3	0.0388	0.0393	0.7	0.4019	0.0037	0.1	0.9212
NREM-Parasomnias	0.0322	0.8	0.3835	0.0765	3.4	0.0672	0.0041	0.1	0.9155
NREM-Parasomnias (probable)	−0.0474	1.7	0.1950	−0.0362	0.7	0.4160	0.0028	0.1	0.9379
SRBD	0.0797	4.3	0.0372	0.1037	4.9	0.0265	−0.0067	−0.2	0.8653
SRBD (probable)	0.0164	0.2	0.6652	−0.0186	0.1	0.7089	0.0593	1.5	0.1247
Circadian rhythm sleep–wake disorders	0.0237	0.4	0.5030	−0.0722	2.0	0.1550	0.0250	0.7	0.4950
Mood disorders	−0.0544	2.1	0.1457	0.0146	0.6	0.4392	−0.1117	−2.9	0.0036
Other mental disorders	−0.0187	0.3	0.6103	0.0342	0.6	0.4392	−0.0271	−0.7	0.4721
Other sleep disorders	−0.0668	3.3	0.0702	−0.0288	0.4	0.5357	0.0910	2.5	0.0138
Somatic illnesses	−0.0425	1.3	0.2561	−0.0412	0.6	0.4401	0.0265	0.7	0.4682
No sleep disorder	0.0245	0.5	0.4810	−0.5591	0.0	0.9816	0.0232	0.6	0.5234
Arousal Predisposition Scale (APS)	0.1537	13.4	0.0002	0.1934	12.8	0.0002 ^3^	−0.1580	−3.7	0.0001 ^3^
Metacognitions-Insomnia Ques. (MCQ-I20)	−0.0591	2.2	0.1345	0.0899	3.2	0.0361 ^3^	−0.0939	−2.3	0.0100 ^3^
	R^2^ = 0.1962, *n* = 845			R^2^ = 0.3402, *n* = 838			R^2^ = 0.2259, *n* = 608		

SE = standardized estimates, ^1^ ordinal regression, ^2^ parametric regression, ^3^ one-tailed.

## Data Availability

The data presented in this study are available on request from the corresponding author. The data are not publicly available due to being clinical data.

## References

[1] Ong J.C., Ulmer C.S., Manber R. (2012). Improving sleep with mindfulness and acceptance: A metacognitive model of insomnia. Behav. Res. Ther..

[2] Waine J., Broomfield N.M., Banham S., Espie C.A. (2009). Metacognitive beliefs in primary insomnia: Developing and validating the Metacognitions Questionnaire–Insomnia (MCQ-I). J. Behav. Ther. Exp. Psychiatry.

[3] Palagini L., Mauri M., Dell’Osso L., Riemann D., Drake C.L. (2016). Trait- and pre-sleep-state-dependent arousal in insomnia disorders: What role may sleep reactivity and sleep-related metacognitions play? A pilot study. Sleep Med..

[4] Palagini L., Piarulli A., Menicucci D., Cheli E., Lai E., Bergamasco M., Mauri M., Kyle S., Espie C., Gemignani A. (2014). Metacognitive beliefs relate specifically to sleep quality in primary insomnia: A pilot study. Sleep Med..

[5] Schredl M., Schackert M., Feld G.B., Schilling C. (2021). Ein Fragebogen zur Erfassung von schlafbezogenen Metakognitionen: Deutsche Kurzform des MCQ-I. Somnologie.

[6] Schredl M. (2003). Continuity between waking and dreaming: A proposal for a mathematical model. Sleep Hypn..

[7] Feige B., Nanovska S., Baglioni C., Bier B., Cabrera L., Diemers S., Quellmalz M., Siegel M., Xeni I., Szentkiralyi A. (2018). Insomnia—perchance a dream? Results from a NREM/REM sleep awakening study in good sleepers and patients with insomnia. Sleep.

[8] Pérusse A.D., De Koninck J., Pedneault-Drolet M., Ellis J.G., Bastien C.H. (2016). REM dream activity of insomnia sufferers: A systematic comparison with good sleepers. Sleep Med..

[9] Schredl M., Schäfer G., Weber B., Heuser I. (1998). Dreaming and insomnia: Dream recall and dream content of patients with insomnia. J. Sleep Res..

[10] Schredl M. (2009). Nightmare frequency in patients with primary insomnia. Int. J. Dream Res..

[11] Levin R., Nielsen T.A. (2007). Disturbed dreaming, posttraumatic stress disorder, and affect distress: A review and neurocognitive model. Psychol. Bull..

[12] Wegner D.M., Wenzlaff R.M., Kozak M. (2004). Dream rebound: The return of suppressed thoughts in dreams. Psychol. Sci..

[13] Kröner-Borowik T., Gosch S., Hansen K., Borowik B., Schredl M., Steil R. (2013). The effects of suppressing intrusive thoughts on dream content, dream distress and psychological parameters. J. Sleep Res..

[14] Bryant R.A., Wyzenbeek M., Weinstein J. (2011). Dream rebound of suppressed emotional thoughts: The influence of cognitive load. Conscious. Cogn..

[15] Malinowski J., Carr M., Edwards C., Ingarfill A., Pinto A. (2019). The effects of dream rebound: Evidence for emotion-processing theories of dreaming. J. Sleep Res..

[16] Wegner D.M., Zanakos S. (1994). Chronic Thought Suppression. J. Personal..

[17] Malinowski J.E. (2015). Dreaming and personality: Wake-dream continuity, thought suppression, and the Big Five Inventory. Conscious. Cogn..

[18] Malinowski J. (2017). High thought suppressors dream more of their negative waking-life experiences than low thought suppressors. Dreaming.

[19] Harvey A.G. (2003). The Attempted Suppression of Presleep Cognitive Activity in Insomnia. Cogn. Ther. Res..

[20] Wang J., Feng X., Shen H. (2022). A presleep consideration of an intrusive thought enhances the possibility of dreaming of it. Dreaming.

[21] Feng X., Wang J. (2022). Presleep Ruminating on Intrusive Thoughts Increased the Possibility of Dreaming of Threatening Events. Front. Psychol..

[22] Goitom A.D., Schredl M. (2020). Dream recall and nightmare frequency in patients with sleep disorders: A diary study. Int. J. Dream Res..

[23] Coren S. (1988). Prediction of insomnia from arousability predisposition scores: Scale development and cross-validation. Behav. Res. Ther..

[24] Coren S. (1990). The arousal predisposition scale: Normative data. BPS.

[25] Clamor A., Warmuth A.M., Lincoln T.M. (2015). Arousal Predisposition as a Vulnerability Indicator for Psychosis: A General Population Online Stress Induction Study. Schizophr. Res. Treat..

[26] Nicassio P.M., Mendlowitz D.R., Fussell J.J., Petras L. (1985). The phenomenology of the pre-sleep state: The development of the pre-sleep arousal scale. Behav. Res. Ther..

[27] Gieselmann A., de Jong-Meyer R., Pietrowsky R. (2012). Kognitive und körperliche Erregung in der Phase vor dem Einschlafen. Z. Klin. Psychol. Psychother..

[28] World Health Organization (1992). The ICD-10 Classification of Mental and Behavioural Disorders: Clinical Descriptions and Diagnostic Guidelines.

[29] Cohen J. (1988). Statistical Power Analysis for the Behavioral Sciences.

[30] Lenhard W., Lenhard A. Berechnung von Effektstärken (Computing Effect Sizes). https://www.psychometrica.de/effektstaerke.html.

[31] Koulack D., Goodenough D.R. (1976). Dream recall and dream recall failure: An arousal-retrieval model. Psychol. Bull..

[32] Palagini L., Ong J.C., Riemann D. (2017). The mediating role of sleep-related metacognitive processes in trait and pre-sleep state hyperarousal in insomnia disorder. J. Psychosom. Res..

[33] Schredl M., Berres S., Klingauf A., Schellhaas S., Göritz A.S. (2014). The Mannheim Dream questionnaire (MADRE): Retest reliability, age and gender effects. Int. J. Dream Res..

[34] Schredl M., Reinhard I. (2008). Gender differences in dream recall: A meta-analysis. J. Sleep Res..

[35] Schredl M., Reinhard I. (2011). Gender differences in nightmare frequency: A meta-analysis. Sleep Med. Rev..

[36] Schredl M., Binder R., Feldmann S., Göder R., Hoppe J., Schmitt J., Schweitzer M., Specht M., Steinig J. (2012). Dreaming in patients with sleep disorders: A multicenter study. Somnologie.

[37] Zadra A.L., Donderi D.C. (2000). Nightmares and bad dreams: Their prevalence and relationship to well-being. J. Abnorm. Psychol..

[38] Aspy D.J., Delfabbro P., Proeve M. (2015). Is dream recall underestimated by retrospective measures and enhanced by keeping a logbook? A review. Conscious. Cogn..

[39] Zunker M., Althoff H.K., Apel J., Lässig H.S., Schültke L., Schredl M. (2015). Comparing questionnaire and diary measures for eliciting nightmare frequency. Int. J. Dream Res..

[40] Wells A. (2009). Metacognitive Therapy for Anxiety and Depression.

[41] Schredl M. (2003). Effects of state and trait factors on nightmare frequency. Eur. Arch. Psychiatry Clin. Neurosci..

[42] Coren S., Mah K.B. (1993). Prediction of physiological arousability: A validation of the Arousal Predisposition Scale. Behav. Res. Ther..

[43] Schredl M. (2018). Researching Dreams: The Fundamentals.

[44] Trauer J.M., Qian M.Y., Doyle J.S., Rajaratnam S.M.W., Cunnington D. (2015). Cognitive Behavioral Therapy for Chronic Insomnia. Ann. Intern. Med..

[45] Galbiati A., Sforza M., Scarpellino A., Salibba A., Leitner C., D’Este G., Mombelli S., Ferini-Strambi L., Castronovo V. (2021). “Thinking About Thinking” in Insomnia Disorder: The Effect of Cognitive-Behavioral Therapy for Insomnia on Sleep-Related Metacognition. Front. Psychol..

